# Mobile Robot Path Planning Based on Ant Colony Algorithm With A^*^ Heuristic Method

**DOI:** 10.3389/fnbot.2019.00015

**Published:** 2019-04-16

**Authors:** Xiaolin Dai, Shuai Long, Zhiwen Zhang, Dawei Gong

**Affiliations:** ^1^School of Mechatronics Engineering, University of Electronic Science and Technology of China, Chengdu, China; ^2^Center of Robot, University of Electronic Science and Technology of China, Chengdu, China

**Keywords:** path planning, ant colony algorithm, A^*^ algorithm, bending suppression, retraction mechanism

## Abstract

This paper proposes an improved ant colony algorithm to achieve efficient searching capabilities of path planning in complicated maps for mobile robot. The improved ant colony algorithm uses the characteristics of A^*^ algorithm and MAX-MIN Ant system. Firstly, the grid environment model is constructed. The evaluation function of A^*^ algorithm and the bending suppression operator are introduced to improve the heuristic information of the Ant colony algorithm, which can accelerate the convergence speed and increase the smoothness of the global path. Secondly, the retraction mechanism is introduced to solve the deadlock problem. Then the MAX-MIN ant system is transformed into local diffusion pheromone and only the best solution from iteration trials can be added to pheromone update. And, strengths of the pheromone trails are effectively limited for avoiding premature convergence of search. This gives an effective improvement and high performance to ACO in complex tunnel, trough and baffle maps and gives a better result as compare to traditional versions of ACO. The simulation results show that the improved ant colony algorithm is more effective and faster.

## Introduction

Path planning is a key issue in the field of mobile robot research. Its main purpose is to find an optimal or suboptimal, safe and collision-free path from the starting point to the target point in the environment with obstacle (Cheng et al., 2010; Deepak et al., 2012; Zhou et al., 2013). According to the degree of intelligence in the process of path planning, mobile robot path planning can be divided into traditional path planning and intelligent path planning. The traditional path planning algorithm includes simulated annealing algorithm (Miao and Tian, 2013), potential function theory (Cetin and Yilmaz, 2014; Nair et al., 2015), fuzzy logic algorithm (Li et al., 2013; Jiang and Li, 2014; Bakdi et al., 2016) and so on. However, these traditional methods can't be further improved in path search efficiency and path optimization. Intelligent path planning algorithm includes Ant Colony Optimization (ACO) (Jovanovic et al., 2016; Wang et al., 2016), genetic algorithm (Arantes et al., 2017; Lin et al., 2017), neural network (He et al., 2016a, 2017a,b) and particle swarm algorithm (Das et al., 2016; Song et al., 2016) and so on. The ant colony algorithm has the advantages of strong robustness, good global optimization ability and inherent parallelism. Moreover, it easily combines with multiple heuristic algorithms to improve the performance of algorithms. So it is widely used in path planning.

However, due to the randomness of probabilistic transfer and the inappropriateness of pheromone intensity update, the traditional ACO will easily fall into the local optimum and tend to poor convergence. To this end, many scholars delivered a variety of improved methods to solve problems regarding pheromone update and path search strategy (Stützle and Hoos, 2000; Zeng et al., 2016; Zhao et al., 2016; Zhang et al., 2017). In Stützle and Hoos (2000), an Ant Colony System (ACS) algorithm was proposed to speed up the convergence rate of ACO by updating pheromones on the path of the optimal ant of each generation. In Zhao et al. (2016), by adaptively changing the volatilization rate and adjusting the pheromone updating formula, the search ability of the ant colony and the convergence rate of the algorithm were improved. In Zhao et al. (2016), some intelligent algorithm was proposed to generate an initial path, which can be transformed into the initial pheromone distribution to avoid blind search of ant colony. In Zhang et al. (2017), the path information (such as the crowded path and the steep path weight) was added into the initial pheromone matrix, which could affects the efficiency of the algorithm. In Zhao et al. (2016), the heuristic function was adjusted to improve the convergence rate of the algorithm according to the target point. In Zeng et al. (2016), it unlimited step length of finding optimal path so that the improved ACO could find a shorter path and its convergence was better. In addition, many scholars have combined the ant colony algorithm with other (intelligent) algorithms (He et al., 2016b; Liu et al., 2016; Yen and Cheng, 2016; He and Zhang, 2017) to improve the convergence rate and the smooth of path. In Liu et al. (2016), the geometric method was used to optimize path. Also in Liu et al. (2016), the force factor in the artificial potential field method is transformed into local diffusion pheromone to improve the ability of the ant colony algorithm to find the obstacle. In Yen and Cheng (2016), the fuzzy ant colony optimization method was proposed to minimize the iterative learning error.

In this paper, an effective version of ant colony algorithm is achieved. It utilizes the evaluation function of A^*^ algorithm to improve the heuristic information of Ant colony algorithm, which accelerates the convergence speed during the search. And MAX–MIN Ant System is used to make the global search ability better by updating the path pheromone of the optimal network. At the same time, the bending suppression operator is introduced to improve heuristic information, which aims to optimize the smoothness of the path. The problem of deadlock is solved by using the retraction mechanism. All these procedures not only give an effective improvement and better performance to ACO, but also give the best results as compare to traditional versions of the algorithm (Zhao et al., 2016) and ACO in complex tunnel, trough and baffle maps. The simulation results show that the proposed algorithm is effective and fast.

## Materials and Methods

### Environment Model

**Figure 1:**
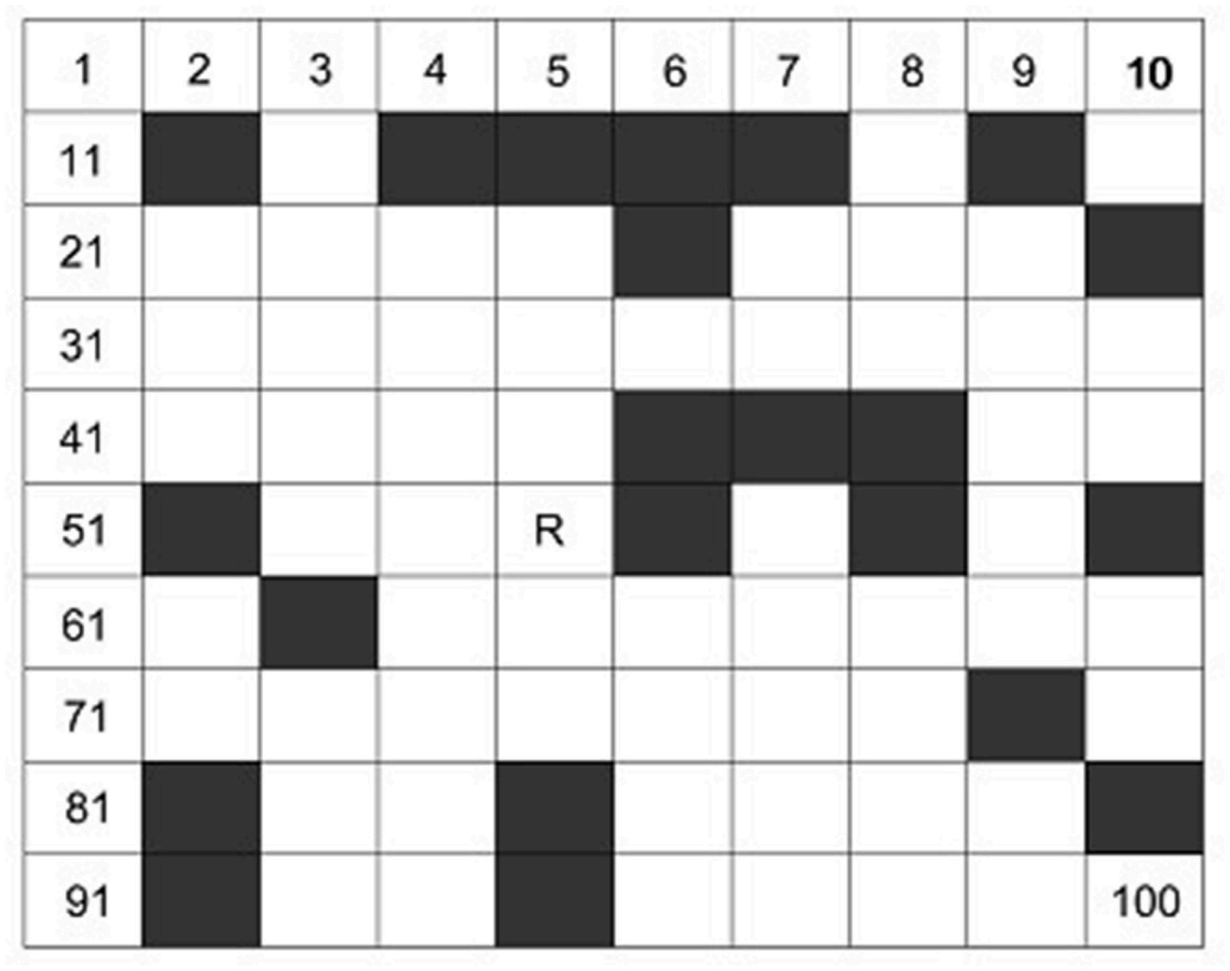
Environment model.

The work environment is built by using the grid model, which divides the robot working space into N^*^N squares. As shown in Figure 1, the gray grids are represented as obstacles (the grid with barriers) and the white grids are represented as free grid squares (the robot can move). In order to identify obstacles, the white grid cell is represented by 0 and the gray grid unit is represented by 1. The grid method is simple and effective to create and maintain grid model. Moreover, the grid method have strong adaptability for obstacle. This method is convenient for computer storage and processing.

The grid model was placed into two-dimensional coordinate system. And then serial number method is adopted to mark each grid. In N^*^N grid map, the starting node is named after *Start* and the target node is named after *Goal*. The position coordinates (*x, y*) corresponding to any grid whose grid number is *R* as follow:

(1) $$\{\begin{array}{l} x=\{\begin{array}{ll} mod\left(R,N\right)-0.5 & if\;mod\;\left(R,N\right)!=0\;\\ N+mod\left(R,N\right)-0.5 & otherwise\; \end{array}\\ y=N+0.5-ceil\left(\frac{R}{N}\right) \end{array}$$

Where *mod* is the surplus operation, *ceil* rounds the elements to the nearest integers toward infinity.

### Ant Colony Algorithm

#### Heuristic Strategy With Direction Information

In the traditional ACO, the probability of the next node is selected by roulette wheel method as follows:

(2) $$P_{ij}^{k}\left(t\right)=\{\begin{array}{ll} \;\frac{{\left(\tau_{ij}\left(t\right)\right)}^{\alpha }\cdot {\left(\eta_{ij}\left(t\right)\right)}^{\beta }}{\sum_{s\in allow_{k}}{\left(\tau_{is}\left(t\right)\right)}^{\alpha }\cdot {\left(\eta_{is}\left(t\right)\right)}^{\beta }} & \;s\in allow_{k}\\ \;0\; & s\notin allow_{k} \end{array}$$

$$\begin{array}{l} {\;\eta }_{ij}\left(t\right)=\frac{1}{d_{ij}}\\ d_{ij}=\sqrt{{\left(x_{j}-x_{i}\right)}^{2}+{\left(y_{j}-y_{i}\right)}^{2}} \end{array}$$

Where τ_*ij*_ is the pheromone trail of the path grid *i* to grid *j*, and η_*ij*_ is the heuristic information of the path grid *i* to grid *j*. α* is* the stimulating factor of pheromone concentration which determine the relative influence of the pheromone trail. *β* is the stimulating factor of visibility which determine the relative influence of the heuristic information. *d*_*ij*_ is the distance between node *i* and node *j*. (*x*_*i*_, *y*_*i*_) and (*x*_*j*_, *y*_*j*_) is the coordinates of grid *i* and grid *j*. *allow*_*k*_ is the collection of grids which ants can choose when ants in the grid *i* (in other words, they are the grids around the grid *i* except the obstacle grid and taboo grid).

#### Coverage and Updating Strategy

According to the traditional ACO, the next node is decided by the roulette wheel method and it is repeated until the target point is obtained. After each iteration is completed, pheromone trails are updated in line with the length of path planning. For each trial during pheromone update, all imperfect pheromones evaporates and only the best pheromones are updated to trials history, because it enables ants to neglect all substandard pheromone trails and improve its coverage efficiency to find a shorter path. Formula (3) is used to update the pheromone quantity on each vertex at the end of each cycle:

(3) $$\{\begin{array}{l} \tau_{ij}\;=\left(1-\rho \right)\tau_{ij}+\Delta \tau_{ij}\\ \Delta \tau_{ij}=\sum_{k=1}^{m}\Delta \text{​​ }\tau_{ij}^{k}\;,\;0<\rho <1\;\; \end{array}$$

where *m* is the number of ants. ρ is pheromone evaporation rate. ${\Delta \tau }_{ij}^{k}$ represents the value of pheromone that the ant *k* leaves in the path of grid *i* to grid *j*. This article uses the ant-cycle-system model, and $\Delta \tau_{ij}^{k}$ is defined as follows:

(4) $$\text{​​}\Delta \text{​​ }\tau_{ij}^{k}\left(k\right)=\{\begin{array}{ll} Q_{1}/L_{k}\left(t\right) & if\;arc\;\left(i,j\right)\;is\;used\;by\;k\;in\;iteration\;t\\ 0 & otherwise \end{array}$$

Where *Q*_1_ is a constant. *L*_*K*_(*t*) is the length of the path that the ant *k* is looking for.

### Improved Ant Colony Algorithm

The traditional ACO has the following shortcomings: Due to the lack of initial pheromone and the unapparent difference of the heuristic value between adjacent grids, it usually requires a longer search time, which leads to the slow convergence rate. When grid model is complex, the robot maybe fall into a deadlock state in which the robot cannot move to the surrounding grids. In the grid map, the path planning with traditional ACO may have more bending times and big cumulative bending angle. To solve the above problems, this paper makes the following improvements.

#### Heuristic Information Based on A^*^ Algorithm

A^*^ algorithm (Duchon et al., 2014) is the most effective direct search method for solving the shortest path in static road network. It is developed on the basis of Dijkstra algorithm, which can avoid blind search to improve search efficiency. In this paper, A^*^ algorithm is used as the heuristic information of path searching to improve the convergence speed of the algorithm and obtain the better path. The bending suppression operator is added to the heuristic information to reduce bending times and cumulative bending angle.

The heuristic cost of A^*^ algorithm is expressed by the estimated function, and the estimated function equation *f*(*n*) is as follows:

(5) $$\begin{array}{lll} f\left(n\right) & = & g\left(n\right)+h\left(n\right) \end{array}$$

$$\begin{array}{lll} h\left(n\right) & = & {\left({\left(n_{x}-g_{x}\right)}^{2}+{\left(n_{y}-g_{y}\right)}^{2}\right)}^{1/2}\\ g\left(n\right) & = & {\left({\left(n_{x}-s_{x}\right)}^{2}+{\left(n_{y}-s_{y}\right)}^{2}\right)}^{1/2} \end{array}$$

where *g*(*n*) is the minimum cost from the source node to the current node. *h*(*n*) is the minimum cost from the current node to the destination node. *n*_*x*_ and *n*_*y*_ are the coordinates of the current node *n* . *g*_*x*_ and *g*_*y*_ are the coordinates of the target node *g*, *s*_*x*_, and *s*_*y*_ are the coordinates of the initial node *s*.

The estimated function of A^*^ algorithm is used as heuristic information to search for global optimal path in ant colony algorithm, and the bending suppression operator is added to the heuristic value of ant colony algorithm to reduce the number of bending times and the large cumulative turning angle. The improved heuristic information formula is as follows:

(6) $$\begin{array}{lll} \eta_{ij}\left(t\right) & = & \frac{Q_{2}}{h\left(n\right)\;+\;g\left(n\right)\;+\;cost\left(bend\right)} \end{array}$$

$$\begin{array}{lll} cost\left(bend\right) & = & \varphi \cdot turn+\psi \cdot thita \end{array}$$

where *Q*_2_is a constant more than 1. *cost*(*bend*) is a bending suppression operator. *turn* is the number of turns from node *n*−1(previous node) to node *n*+1 (next node). *thita* is the angle between the line segment of node *n*−1 to node *n* (current node) and the line segment of node *n* to node *n*+1. φ is the coefficient converting turning times into grid length. ψ is the coefficient converting angle into grid length.

#### Solve the Deadlock Problem

**Figure 2:**
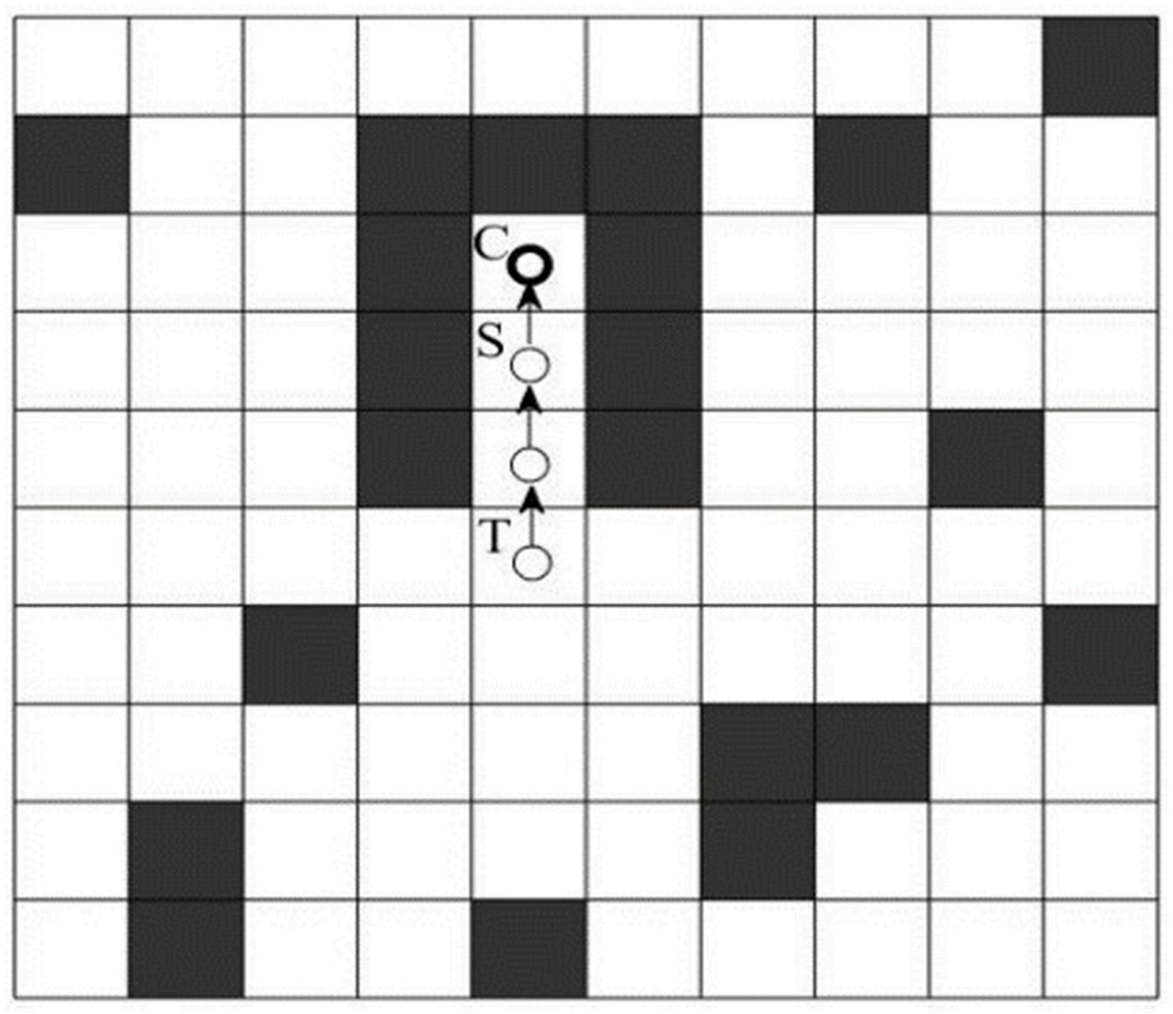
Deadlock state diagram.

When the robot environment is more complex (especially the ants go into the environment of concave obstacles), due to the presence of the taboo table, the ants may fall into a deadlock state without the next grid to move. As shown in Figure 2, when the ant travels from the grid *T* to the grid *S*, the next optional grid is *C*. At this time, the ant is trapped in a deadlock state and it cannot move to its surrounding grid.

For the deadlock problem, Wang and Yu (2011) adopted the early death strategy, which deleted the ants trapped in a deadlock state from the ant colony and did not update the global pheromone. However, when more of the ants are trapped in the deadlock state, the number of ants that can reach the goal is significantly reduced, which results in a decrease in the diversity of solutions and is not conducive to the search of optimal path for ants. In this paper, the improvement measure is that the ants adopt retraction mechanism when they fall into the deadlock state. As shown in Figure 2, the ant, which has walked into the grid, is trapped in the deadlock state, and the improved strategy allows the ant to roll back one step and updates the taboo table information. If the ant is still trapped into a deadlock state, the ant will continue to rollback untill grid *T*. At this moment, the ant escapes the deadlock area. Since the deadlock edge may be the part of global optimal path, no pheromone punishment is carried out on the deadlock edge. The retraction mechanism cannot prevent ants from entering a deadlock state, but it lets the deadlocked ants return back to the previous grid until there is a feasible grid around the ants, so the ACO with the retraction mechanism has higher efficiency and fewer iterations. The ACO with the retraction mechanism and without the retraction mechanism is compared in section The Retraction Mechanism Results Analysis below.

#### Max–Min Ant System

As the traditional ant colony algorithm may cause premature convergence and precocious phenomenon, it needs to improve algorithm to solve these problem. The MAX-MIN Ant System (MMAS) (Stützle and Hoos, 2000) can solve these problems well.

*(1) Pheromone trail updating*. After each iteration trial, the pheromone is submitted into update history in traditional ant colony algorithm. While in the MMAS, only the path pheromone of the optimal network is updated after the iteration is completed. Accordingly, the modified pheromone trail update rule is stated by:

(7) $$\begin{array}{lll} \tau_{ij}\left(t+1\right) & = & \left(1-\rho \right)\tau_{ij}\left(t\right)+{\Delta \tau }_{ij}^{best} \end{array}$$

$$\begin{array}{l} {\Delta \tau }_{ij}^{k}\left(t\right)=\frac{Q_{1}}{L^{best}}+\frac{Q_{3}}{C_{turn}^{best}}\\ C_{turn}^{best}=\omega_{1}Cals\left(l\right)+\omega_{2}Turns\left(l\right)\\ \omega_{1}=\frac{V_{robot}}{W_{robot}}\\ \omega_{2}=V_{robot}\times t_{a} \end{array}$$

where *Q*_3_ is a constant more than 1.*L*^*best*^ denotes to the shortest path currently found by the algorithm. *Cals*(*l*) represents the sum of all the angles of turning on the best optimized path. *Turns*(*l*) is the sum of the turns on the best optimized path. *w*_1_ and *w*_2_ represent different weight coefficient and are set by analyzing the robot's structure and kinematics (Wu et al., 2013; Li et al., 2017). The *w*_1_ and *w*_2_ can convert turning angle and turning times into grid length, respectively. *V*_*robot*_ represents the constant speed of a mobile robot. *W*_*robot*_ represents the angular speed of a mobile robot as it turns. *t*_*a*_ represents the time of acceleration and deceleration as the mobile robot turns once.

*(2) Pheromone trail limits*. In order to avoid the situation that the traditional ant colony algorithm may falls into local optimal solution and loses the further search space ability by pheromone accumulation, the pheromone trail of the MMAS is limited in the upper limits and lower limits [*T*au_*min*_,*T*au_*max*_,]. The formula is:

(8) $$\text{Tau}=\{\begin{array}{ll} {\text{Tau}}_{min}, & \text{ Tau}\leq {\text{Tau}}_{min}\;\\ \text{Tau}, & {\text{Tau}}_{min}<\text{Tau}\leq {\text{Tau}}_{max}\\ {\text{Tau}}_{max}, & \text{ Tau}>{\text{Tau}}_{min}\; \end{array}$$

### Aco Procedure

To sum up, specific steps of mobile robot path planning based on the improved ant colony algorithm are as follows:

*Step 1:* The working environment is modeled by the grid method, and the starting point *start* and the target point *goal* of the mobile robot are given.
*Step 2:* Initialize the ant system. Set the number *m* of ants, parameter α which determines the relative influence of the pheromone trail, parameter β which determines the heuristic value, the global pheromone volatilization coefficient ρ, pheromone intensity *Q*_1_ and other related parameters.
*Step 3:* Update taboo table. Place the ant *k* (*k* = 1, 2, ⋯ , *m*) on the current node and add the current node to the corresponding taboo table.
*Step 4:* Process deadlock. According to the taboo table, it will judge whether ants are trapped in a deadlock state. If the ants are in a deadlock state, the retraction mechanism will be adopted and the deadlock node will be added to the taboo table. Conversely, it will judge whether the ants reach the target point. If the ants reach the target point, it will turn to Step 6, otherwise it will turn to Step 5.
*Step 5:* Select the next grid. It will calculate the heuristic function according to formula (6), and calculate the probability function according to formula (2). Finally, it will use the roulette method to select the next feasible grid. If the ants reach the target grid, it will turn to Step 6, otherwise it will turn to Step 3.
*Step 6:* If the ants reach the target node, it will repeat Step 3 until each ant completes the search target during its iteration process and then turn to Step 7.
*Step 7:* Update pheromone. After each iteration, if the number of iterations satisfies inequality *N* ≤ *N*_max_, it will update the path pheromone and determine whether it meets the convergence conditions. If it meets the convergence conditions, it will withdraw. If it does not meet, it will turn to Step 3. If the number of iterations satisfies inequality *N* > *N*_max_, it will be not counted further. The final result is output as long as the end condition is satisfied.

The implementation process of improved ant colony algorithm is as in Table 1.

**Table 1:** Description of ACO algorithm for solving path planning.

**Algorithm** A*MMAS
**Begin**
create grid environment
initialize the ant colony system
**Repeat**
**for** each ant k **do**
**if** grid *i* ∈ *allow*_*k*_ **then**
**if** grid *i* ∈ *taboo*_*deadlock*_ **then**
fallback
**end if**
according to formula (2) and (6) select next grid j
Update taboo
**end if**
Update pheromone on each iteration by improved MMAS method according to formula (7) and (8)
**Until** algorithm convergence
**Return** best grid serial number
**END**

## Results

In order to verify the effectiveness of the improved ant colony algorithm, this paper uses MATLAB software to simulate. It is more convincing to use comparative method to carry out experiments under the same experimental conditions. In the simulation, the main parameter values of the ACO should be determined firstly. The main parameters include number of ants, stimulating factor of pheromone concentration, stimulating factor of visibility and pheromone evaporation coefficient. Through parameter analysis method (Wu et al., 2010), the relationship between each parameter and simulation results (path length, number of iterations) can be obtained. According to the relationship between each parameter and simulation results (Shi et al., 2014), we can get the value of the main parameters in the ACO. In the simulation, value of each parameter in the ACO is as in Table 2:

**Table 2:** Values of the main parameters in the ACO.

**Number of ants *m***	**Stimulating factor of pheromone concentration **α****	**Stimulating factor of visibility **β****	**Pheromone evaporation coefficient **ρ****	**Pheromone intensity *Q***
50	1	5	0.5	10

### Comparative Analysis of Path Planning Algorithms

The experiment was divided into four parts according to four types of maps(the common map, the tunnel map, the trough map and the baffle map) and three algorithms (the traditional ant colony algorithm, the algorithm (Zhao et al., 2016) and the improved ant colony algorithm proposed in this paper) are simulated on each map in turn. The convergence speed, shortest path length and bending suppression effect of those algorithms are compared.

**Figure 3:**
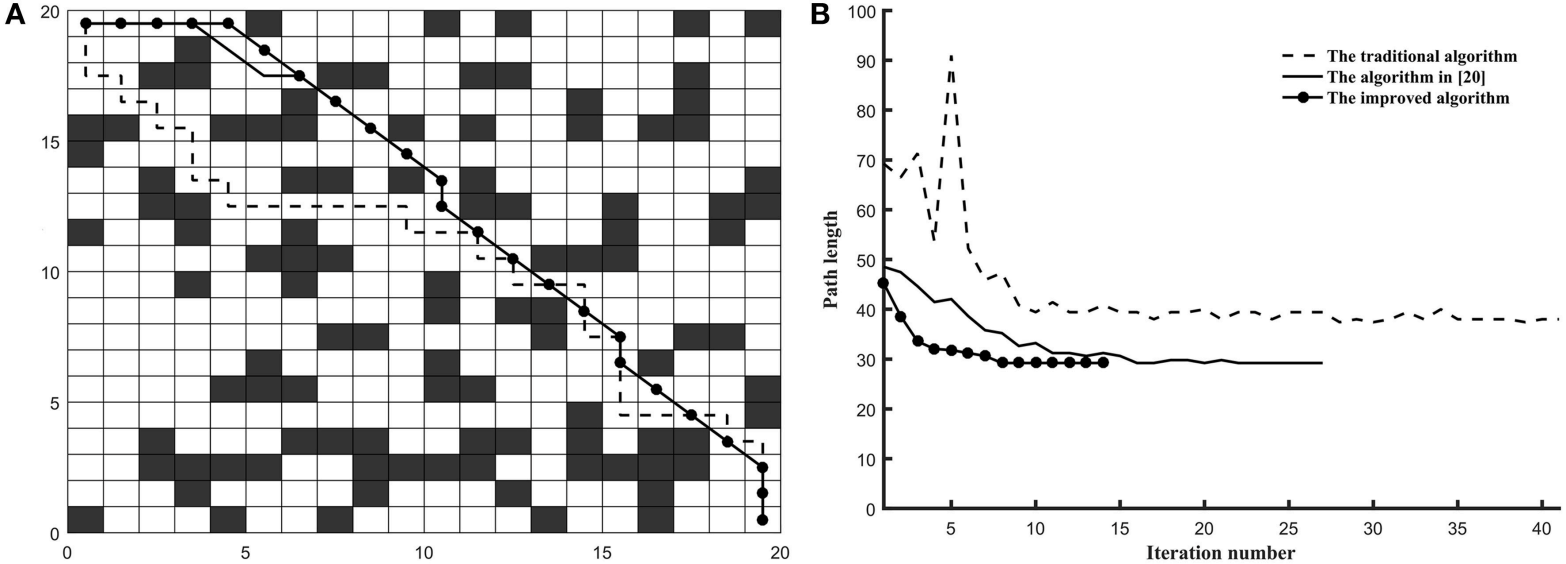
The test results of three algorithms run on common map. **(A)** Simulation results in 20*20 grid. **(B)** Convergence curve.

**Figure 4:**
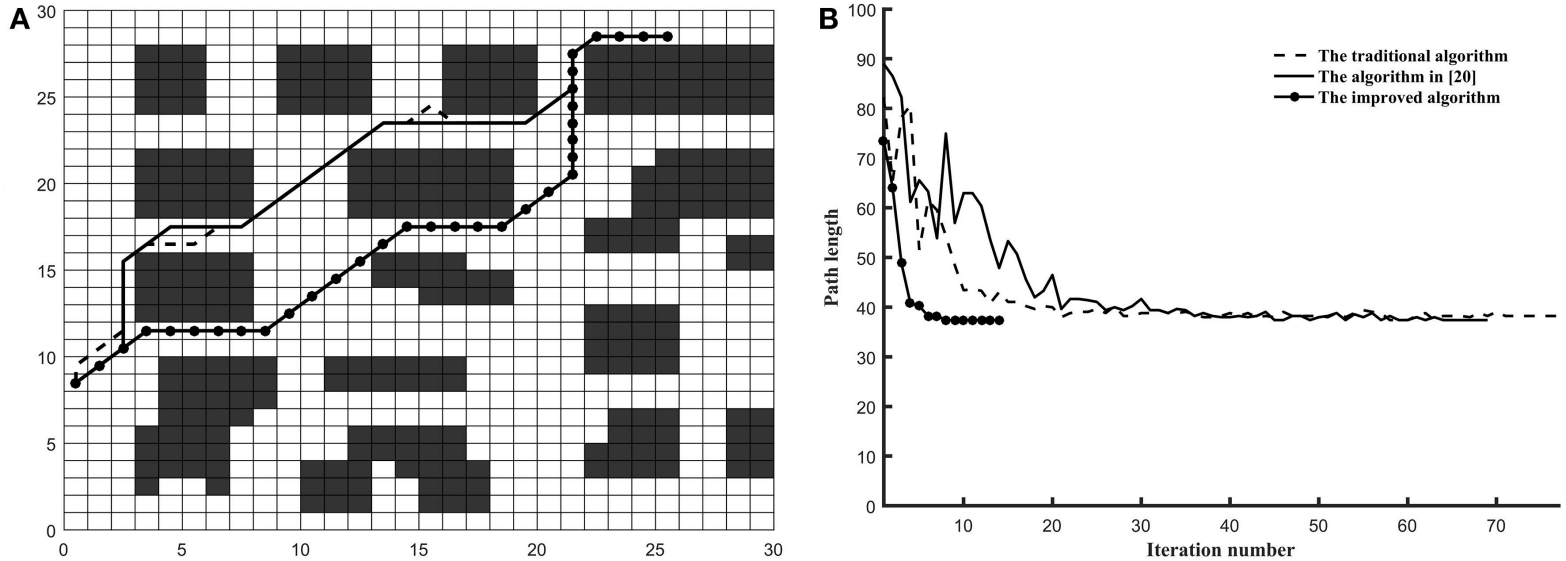
The test results of three algorithms run on tunnel map. **(A)** Simulation results in 30*30 grid. **(B)** Convergence curve.

**Figure 5:**
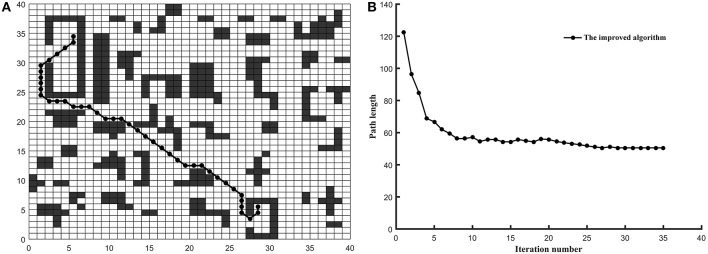
The test results of three algorithms run on trough map. **(A)** Simulation results in 40*40 grid. **(B)** Convergence curve. (Other two algorithms is failed in trough map).

**Figure 6:**
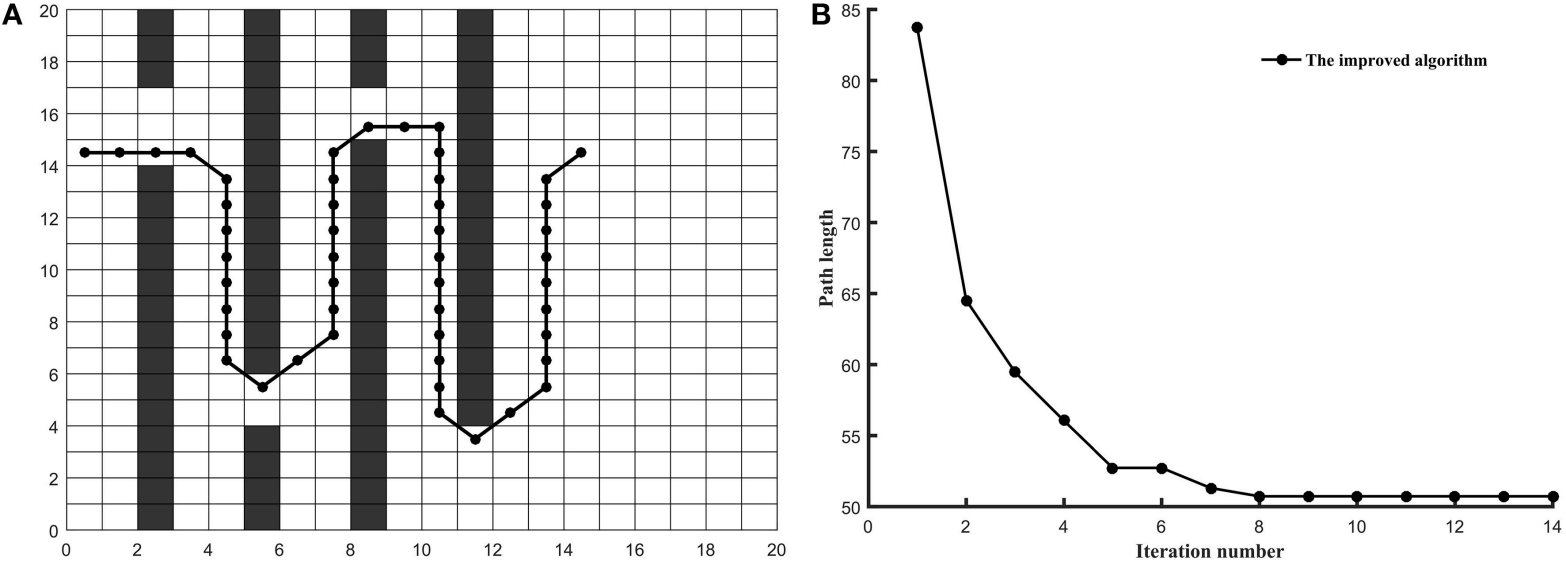
The test results of three algorithms run on baffle map. **(A)** Simulation results in 20*20 grid. **(B)** Convergence curve. (Other two algorithms is failed in trough map).

1. *Example 1*. In this example, the environment of the robot was built into the 20^*^20 grid model and the three algorithms are tested on the common map. The coordinates of grid *Start* and grid *Goal* is (0.5, 19.5) and (19.5, 0.5) (shown in Figure 3), respectively.
2. *Example 2*. In this example, the environment of the robot was built into the 30^*^30 grid model and the three algorithms are tested on the tunnel map. The coordinates of grid *Start* and grid *Goal* is (0.5, 8.5) and (15.5, 18.5) (shown in Figure 4), respectively.
3. *Example 3*. In this example, the environment of the robot was built into the 40^*^40 grid model and the three algorithms are tested on the trough map. The coordinates of grid *Start* and grid *Goal* is (5.5, 34.5) and (28.5, 5.5) (shown in Figure 5), respectively.
4. *Example 4*. In this example, the environment of the robot was built into the 20^*^20 grid model and the three algorithms are tested on the baffle map. The coordinates of grid *Start* and grid *Goal* is (0.5, 14.5) and (14.5, 14.5) (shown in Figure 6), respectively.

As shown in Figure 3, the optimized path length of the improved ant colony algorithm is 29.2133 and the number of bending times is 6. The improved ant colony algorithm is basically as same as the path planning effect of the ant colony algorithm (Zhao et al., 2016) on the path length, but it is 25% lower on bending times than the ant colony algorithm (Zhao et al., 2016). Compared with the traditional ant colony algorithm, it is 73% reduction in the number of bending times.

As shown in Figure 4, the optimized length of the improved ant colony algorithm is 37.3849, and the number of bending times is 7. In the shortest path length, the improved ant colony algorithm is basically as same as the algorithm (Zhao et al., 2016). In the number of bending times, it is 50% decrease than the traditional ant colony algorithm and is 22% decrease than the algorithm (Zhao et al., 2016).

As shown in Figure 5, the optimized path length of the improved ant colony algorithm is 51.1128. In Figure 6, the optimized path length of the improved ant colony algorithm is 50.7280. But in Figures 5, 6, both the traditional ant colony algorithm and the algorithm (Zhao et al., 2016) can't search the global optimized path. Even as the scale of the problem expands and the environment map becomes more and more complex, the improved algorithm can still perform very well.

The results of the three algorithms that run 100 times in same map environments are shown in Table 3. Compared with the traditional ant colony algorithm and the algorithm (Zhao et al., 2016), the improved algorithm has a good performance on the efficiency. At the same time, it has a good adaptability in a complicated area. The improved algorithm proposed in this paper can be used not only in the path planning of mobile robots, but also in the path planning of robot manipulators (Yang et al., 2017, 2018).

**Table 3:** Test results for three algorithms under different maps.

**Map**	**Algorithm**	**Optimal solution of the algorithm**	**The average of the shortest distance**	**Average iteration times**	**Average time-consuming(sec)**	**Number of bends**
Common map	①	37.4143	38.6335	40	9.22	22
	②	29.2133	29.4506	33	7.26	10
	③	29.2133	29.3807	12	4.89	10
Tunnel map	①	38.2133	38.6325	47	26.92	17
	②	37.3849	38.4813	35	20.62	12
	③	37.3849	38.1262	16	17.97	10
Trough map	①	–	–	–	–	–
	②	–	–	–	–	–
	③	51.1128	51.8471	40	88.20	13
Baffle map	①	–	–	–	–	–
	②	–	–	–	–	–
	③	50.7280	51.0605	15	8.40	13

*①: The traditional ant colony algorithm*.

*②: The algorithm [20]*.

*③: the improved ant colony algorithm*.

### The Retraction Mechanism Results Analysis

In order to show the function of retraction mechanism, the ACO with the retraction mechanism and ACO without the retraction mechanism are tested on the trough map and the baffle map, respectively.

**Figure 7:**
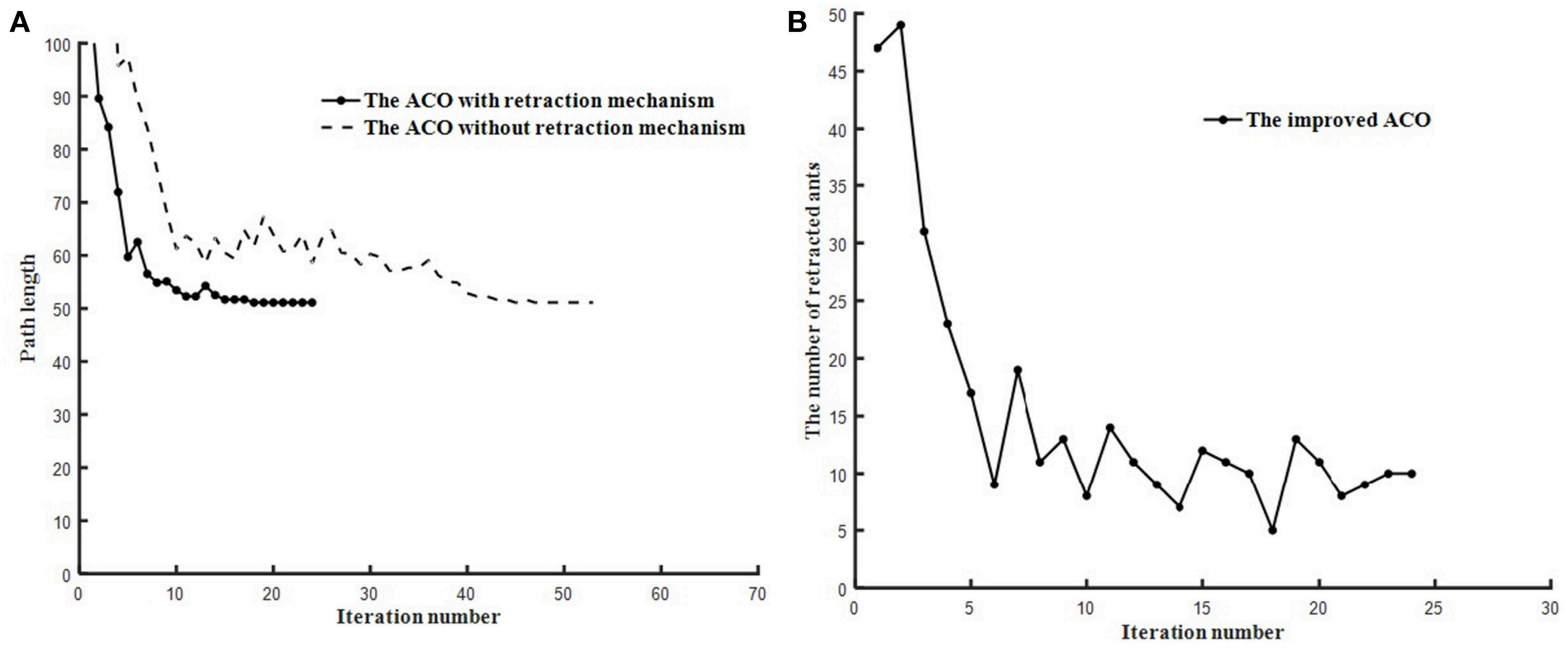
The test results of two algorithms run on trough map. **(A)** Path planning comparison. **(B)** Ant retraction number curve.

**Figure 8:**
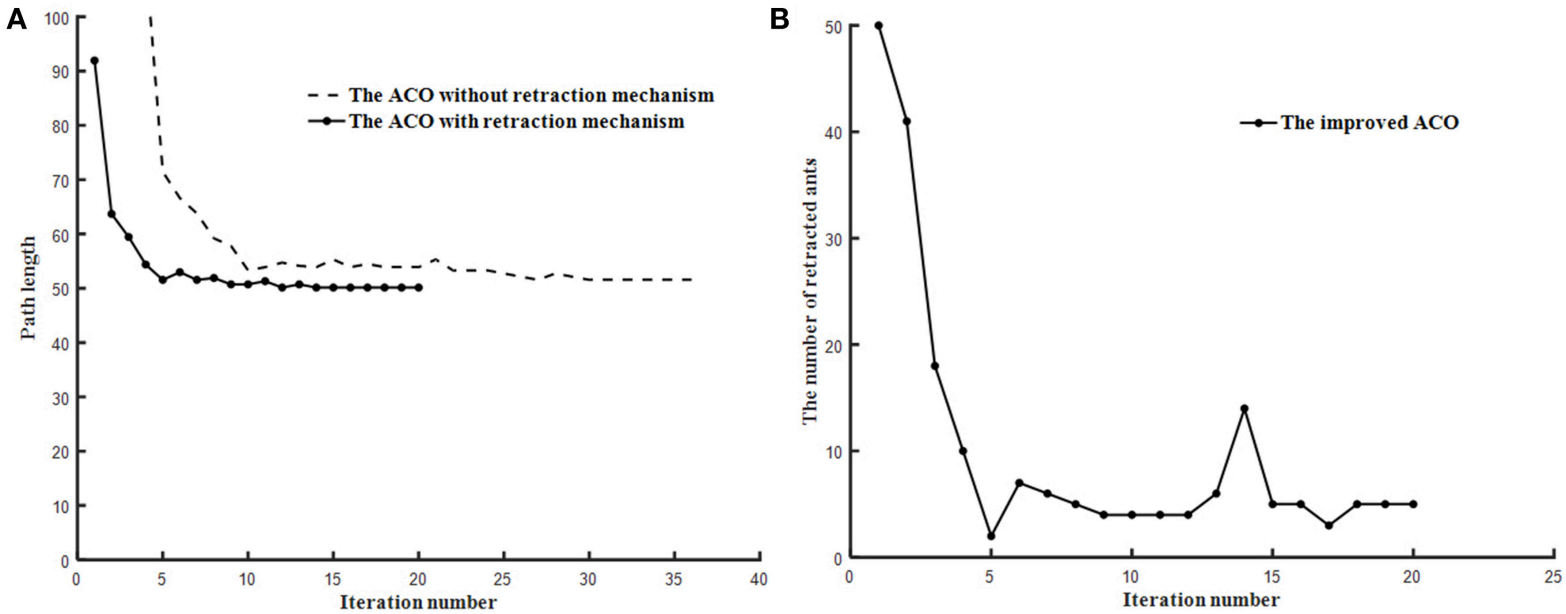
The test results of two algorithms run on baffle map. **(A)** Path planning comparison. **(B)** Ant retraction number curve.

As shown in Figures 7A, 8A, ACO with the retraction mechanism has higher efficiency and fewer iteration than ACO without the retraction mechanism. When ants fall into deadlock state, the retraction mechanism is used to replace the early death strategy, which avoids a large number of ant deaths in one iteration. Therefore, each ant can obtain a path by using the retraction mechanism, which increases the diversity of results and is beneficial to find the optimal path. As shown in Figures 7B, 8B, the number of ant retracted in the initial stage of the algorithm is higher than in the middle and later stage of the algorithm and the retraction mechanism can effectively suppress the decline of the number of ants.

## Discussion

This paper makes a valuable contribution to the improvement of ant colony algorithm in complicated maps for the mobile robot, especially the improvement on convergence speed, shortest path length and bending suppression effect. The estimated function of improved A^*^ algorithm is used as the heuristic function to improve search efficiency and smoothness of path. By employing the retraction mechanism and the improved MAX–MIN Ant System method, the problem of ant deadlock is solved and the global search ability of the algorithm is improved.

Three algorithms are researched on path planning in the common map, tunnel map, trough map and baffle map, respectively. Compared with the traditional ant colony algorithm and the algorithm (Zhao et al., 2016), the improved ant colony algorithm is better in the convergence rate and the bending suppression effect. Compared with the traditional ant colony algorithm, the improved ant colony algorithm has more than 65% reduction in number of iterations and 41% decrease in bending suppression. In addition, the improved ant colony algorithm is 54% lower than the algorithm (Zhao et al., 2016) in number of iterations. To sum up, this paper proves the effectiveness, rapidity and adaptability of the improved ant colony algorithm in the complex map environment.

## Author Contributions

XD and DG proposed the innovation and designed the experiment in this study. ZZ and SL performed the simulation experiment and analyzed the experiment results. XD checked the results. SL wrote the manuscript and DG provided writing advice of manuscript.

### Conflict of Interest Statement

The authors declare that the research was conducted in the absence of any commercial or financial relationships that could be construed as a potential conflict of interest.
